# Titanium-Nitride Coating of Orthopaedic Implants: A Review of the Literature

**DOI:** 10.1155/2015/485975

**Published:** 2015-10-25

**Authors:** Ruud P. van Hove, Inger N. Sierevelt, Barend J. van Royen, Peter A. Nolte

**Affiliations:** ^1^Department of Orthopaedics, Catharina Hospital, 5623 EJ Eindhoven, Netherlands; ^2^Research Centre Linnaeus Institute, Spaarne Hospital, 2134 TM Hoofddorp, Netherlands; ^3^Department of Orthopaedics, VU University Medical Centre, 1081 HZ Amsterdam, Netherlands; ^4^Department of Orthopaedics, Spaarne Hospital, 2134 TM Hoofddorp, Netherlands

## Abstract

Surfaces of medical implants can be enhanced with the favorable properties of titanium-nitride (TiN). In a review of English medical literature, the effects of TiN-coating on orthopaedic implant material in preclinical studies were identified and the influence of these effects on the clinical outcome of TiN-coated orthopaedic implants was explored. The TiN-coating has a positive effect on the biocompatibility and tribological properties of implant surfaces; however, there are several reports of third body wear due to delamination, increased ultrahigh molecular weight polyethylene wear, and cohesive failure of the TiN-coating. This might be due to the coating process. The TiN-coating process should be optimized and standardized for titanium alloy articulating surfaces. The clinical benefit of TiN-coating of CoCrMo knee implant surfaces should be further investigated.

## 1. Introduction

Titanium-nitride (TiN) is a ceramic which has general properties such as great hardness (2000 kg/mm^2^), high decomposition temperature (2949°C), defect structure, that is, deviation from stoichiometry, chemical stability at room-temperature, superconductivity, and a gold-yellow color [1]. TiN can be prepared by direct reaction of titanium or titanium hydrogen powder with nitrogen at 1200°C [1]. Using nitrogen ion implantation, physical vapor deposition, and plasma ion nitriding, titanium surfaces can be enhanced with a TiN layer [2]. Single crystals of TiN can also be vapor deposited on other metals [1]. Recently newer techniques have been introduced for TiN-coating of titanium alloys, such as powder immersion reaction assisted coating (PIRAC) [3], nitrogen plasma immersion ion implantation (PIII) [4], and Hardion+ nitrogen implantation technique [5], to improve the adhesion of the TiN-coating to the implant material.

TiN is mainly used as a coating to enhance other materials with the properties of TiN. TiN showed encouraging blood tolerability properties with a hemolysis percentage near to zero [6]. Therefore, TiN-coatings are used in cardiology for ventricular assist devices for patients with heart failure [7] and for pacemaker leads [8]. In neurology, TiN-coated electrodes are investigated for the development of chronically implanted devices for the treatment of, for example, spinal cord injury [9]. TiN-coating is applied in dentistry to dental implants, because of the excellent biological properties of TiN, such as the reduction of the release of cobalt-chromium-molybdenum (CoCrMo) ions, and the aesthetic aspect of the “golden color” [10, 11].

In 1972, Steinemenan patented the “use of implants of titanium or a titanium alloy for the surgical treatment of bones” [12]. This included a surface layer, for example, nitride, to prevent abrasion and corrosion of the implant and to prevent fretting of contacting implants [12]. In 1997, Buechel and Pappas patented the “prosthesis with biologically inert wear resistant surface” [13]. The load bearing surfaces are coated with biologically inert abrasion resistant material, such as TiN, preferably 8–10 microns thick, harder than the substrate for preventing wear and leaching of ions [13]. Besides the suggested beneficial effect of TiN-coating of the bearing surfaces in cemented and uncemented prosthesis, the TiN-coating might also be beneficial at the bone-implant surface of uncemented prosthesis because it is biologically inert.

Untreated titanium-aluminum-vanadium alloy (Ti6Al4V) showed excessive wear of femoral heads, and surface treatment of Ti6Al4V by TiN-coating was discussed as a possibility to enhance the Ti6Al4V surface [14]. The purpose of this review is to identify the effects of TiN-coating of orthopaedic implant material in preclinical studies and whether these effects influence the clinical outcome of TiN-coated orthopaedic implants.

## 2. Method

This study focuseson preclinical and clinical studies using TiN-coated orthopaedic implants and implant material. Inclusion criteria were clinical studies on TiN-coated orthopaedic implants and preclinical studies on wear and biocompatibility of TiN-coated implant material. To identify all relevant studies on TiN-coated orthopaedic implants and implant material in English scientific literature, the following databases were searched: Medline (1947 to January 2015), Embase Classic and Embase (1947 to January 2015). No restrictions were made to the type of studies. Papers outside the English language, abstracts from scientific meetings, and unpublished reports were excluded. References of retrieved publications were used to add studies meeting the inclusion criteria that were missed by the electronic search. The Medline search is defined in Appendix. The query was checked for inconsistencies using PubMed Query Editor 0.1. Selection of studies was first performed by screening titles and abstracts. In case of insufficient information in the title or abstract, full text copies were retrieved to make a decision for the article selection. References of retrieved publications were used to add studies meeting the inclusion criteria that were missed by the electronic search.

## 3. Results

**Figure 1:**
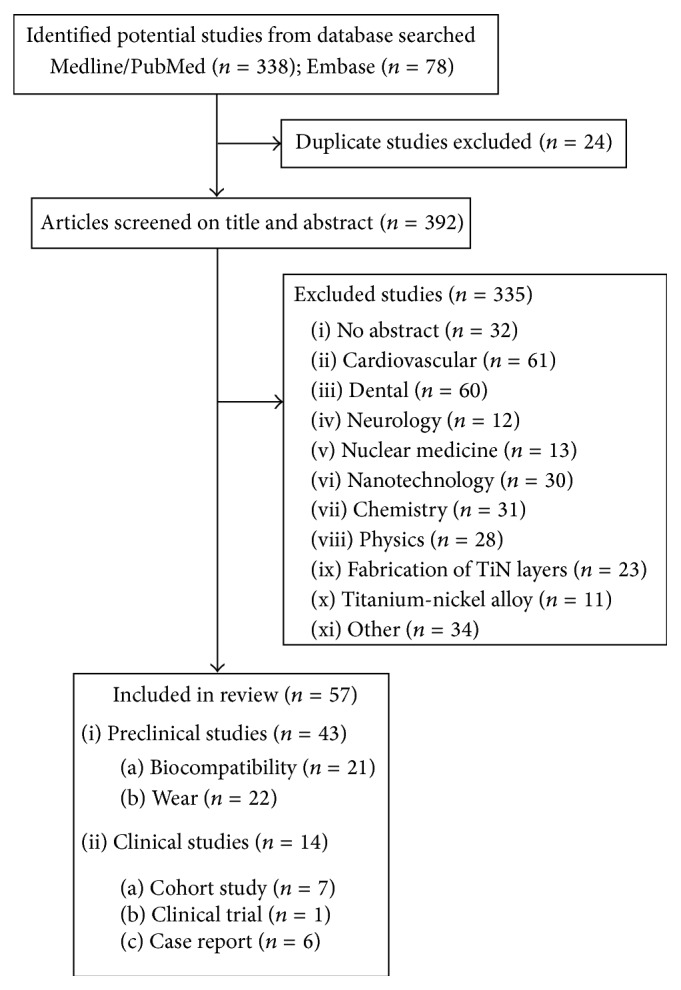
Flow diagram of the search process. Duplicate studies were excluded as well as studies of which there was no abstract. Studies on cardiovascular, dental, and neurological titanium-nitride coated implant material and other studies were excluded. Four studies on dental titanium-nitride coated implant material were included in this study in Section 3.1.

The results of the combined Medline and Embase search are shown in Figure 1. After examining titles and abstracts, a total of 335 out of 394 studies were excluded, due to the absence of abstracts (*n* = 32), research in medical fields other than orthopaedic (61 cardiovascular, 60 dental, 12 neurological, and 13 nuclear medicine), or other reasons (*n* = 157). No additional studies were identified from reference lists of the identified articles.

### 3.1. Preclinical Studies: Biocompatibility

A high variety of cell types were used for cell culture on TiN-coated implant materials: human bone marrow stem cells [15–17], human primary osteoblasts [18], Saos-2 osteoblast-like cells [19], human fibroblasts [20], human fetal osteoblasts [5, 21], U937 macrophages and L929 fibroblasts [22], mouse fibroblasts [23–25], murine monocytes [26], and murine calvarial osteoblasts [4, 27]. Studies on proliferation and differentiation of cells cultured on TiN-coated materials compared with control material are shown in Table 1. In several studies, no difference in proliferation of cells was found on TiN-coated materials compared with their controls [5, 15, 16, 19, 20]; however, an increase in proliferation of cells on TiN-coated materials compared with their controls was observed in other studies [4, 18, 23, 27, 28]. No differences were found on TiN-coated materials compared with their controls in differentiation of cells [15, 16, 18, 19, 27, 28], cell morphology [20, 24, 29], cell adhesion [15, 16, 29, 30], viability [21], and metabolic activity [24]. One study showed a higher number of cells adhered to TiN-coated material compared with the control [17]. Cell viability of cells cultured in the presence of Ti6Al4V, CoCrMo, and TiN-particles decreased after 4 hours to 58%, 44%, and 44%, respectively, but recovered after 24 hours to 78%, 51%, and 65%, respectively [26]. Viability of other cells cultured in the presence of different concentrations of TiN-debris was foremost influenced by the concentration of 50 *μ*m^3^ per cell [22].

TiN-coated materials were implanted in the femurs of dogs [31], rats [3], and rabbits [32]. In dog femurs, some regions of the TiN-coated implants showed no bone opposition at 4 weeks, but from 24 weeks some specimens showed direct bone opposition [31]. The affinity of bone to the implant index of TiN-coated stainless steel was comparable with alumina, but not significantly better than uncoated stainless steel [31]. TiN-coated Ti6Al4V rods were implanted in rat femurs and showed similar biocompatibility and bone-bonding properties compared with uncoated Ti6Al4V [3]. Relative bone area and bone-implant contact of TiN-coated commercially pure (cp) titanium threaded implants in rabbits was similar compared with TiO_2_-coated cp titanium [32].

Results of three independent experiments showed a lower adhesion and proliferation (*P* < 0.05) over 24 hours of bacteria cultures* S. pyogenes* and* S. sanquinis* on TiN-coated titanium plasma sprayed surfaces (TPS) compared with uncoated TPS [16].

### 3.2. Preclinical Studies: Wear

TiN-coated Ti6Al4V showed a high scratch resistance [33, 34] and low coefficient of friction [33, 35], reduction of abrasive particle formation and less ultrahigh molecular weight polyethylene (UHMWPE) wear [35], more resistance to fretting and corrosion [5, 36, 37], reduction of wear [38], lower ion release rates [5, 39], and low fatigue cycle [40] compared with uncoated Ti6Al4V. Studies on wear of TiN-coated materials compared with their controls are shown in Table 2. The TiN-coating of Ti6Al4V showed minor to no signs of surface delamination, surface scratching, or coating failure in simulator tests [35, 37, 41–43]. However, in one study, a high incidence of adhesive coating failure was found in PVD TiN-coated Ti6Al4V, and the TiN-coating was prone to pitting and blistering at small coating defects [44]. Also, the wear behavior is affected heavily by pinholes in the PVD TiN-coating of CoCrMo [45, 46]. In a pin-on-plate test, TiN-coated CoCrMo showed a fourfold increase in wear rate compared with CoCrMo due to catastrophic adhesive failure of the TiN-coating [22]. In a metal-on-UHMWPE hip simulator test, minimal abrasive wear without signs of pitting, delamination, or overheating of the UHMWPE was found when in contact with TiN-coated Ti6Al4V [42]. TiN-coated Ti6Al4V showed lower UHMWPE wear rates compared with Ti6Al4V [35] and 316L stainless steel [41]. TiN-coated CoCrMo showed less adhesion to polyethylene compared with CoCrMo, and CoCrMo showed a catalytic effect on the degradation of polyethylene whereas TiN is inert [46]. Although the volumetric wear rate of UHMWPE was reduced with 42% using a hybrid process for the TiN-film compared with commercialized CoCrMo [47], in another study, the average volumetric wear rate of UHMWPE to TiN-coated CoCrMo was not superior to CoCrMo or alumina [33]. Also, in a multidirectional wear test using UHMWPE specimens, no significant differences were found between TiN-coated CoCrMo and uncoated CoCrMo in coefficient of friction, and wear rate on damaged and undamaged surfaces [48]. Furthermore, friction and wear of the UHMWPE counterface was dependent of the lubricant used [24]. In the presence of protein, wear is very low independently of the surface roughness in the TiN/UHMWPE contact pair [49].

In a metal-on-metal hip simulator test, TiN-coated CoCrMo showed a lower wear rate compared with uncoated CoCrMo [50, 51]. The average wear of the uncoated insert articulating with a TiN-coated femoral head was greater compared with the uncoated femoral heads [51].

### 3.3. Clinical Studies

Clinical studies on survival of TiN-coated orthopaedic implants are presented in Table 3. In a cohort of 76 patients who received a cemented hip prosthesis, 60 received a CoCrMo head and 16 received a TiN-coated titanium head [52]. Loosening of the femoral component occurred in 44.4% of the hips with a TiN-coated titanium head and in 21.6% of the hips with a CoCrMo head (*P* = 0.11) with a 26-month follow-up [52].

In a postmortem retrieval analysis of a cementless TiN-coated Ti6Al4V THA one year after index surgery, the TiN-coated Ti6A14V femoral head showed circular voids without TiN-coating and voids filled with circular droplets of pure titanium [53]. Adhered to the TiN-coating, pure titanium and Ti6Al4V debris was found [53]. In an analysis of four TiN-coated Ti6Al4V femoral heads retrieved at revision after a period of in vivo articulation against UHMWPE liners, TiN-coating breakthrough and fretting occurred in 2 out of 4 retrieved prostheses [54].

Failures of a cementless TiN-coated titanium alloy-on-UHMWPE resurfacing THA were suggested to be due to the use of conventional UHMWPE instead of highly cross-linked UHMWPE [55]. In a case of a failed cementless TiN-coated titanium alloy-on-UHMWPE resurfacing THA eleven years after index surgery, severe wear of the polyethylene liner with erosion of the femoral head into the metal acetabular shell was reported [56].

In the radiological follow-up (16.6 months, range 12–39 months) of 330 hips with a press-fit polyethylene cup with TiN-coated stainless steel mesh (Sulmesh, Sulzer, Winterthur, Switzerland) for the bone-implant interface, there was one case showing a radiolucent line around the cup; however, this was without clinical problems [57]. There was insufficient stability of the mesh in 4 of the 330 cases [57].

In a retrospective study of mainly cementless TiN-coated CoCrMo mobile bearing TKAs, revision surgery was performed in 4.9% [58]. Prosthetic fractures were found in four knees and involved the posteromedial flange of right-sided, size 5, femoral components only [58]. A case report on the fracture of a cementless TiN-coated CoCrMo femoral component met with these findings [59]. Another case, which concerned the fracture of the medial flange posterior to the peg of a TiN-coated Ti6Al4V TKA, was also reported [60].

In a recently published RCT, no differences in postoperative pain, KSS, revision surgery, knee flexion and knee flexion contracture, knee circumference, and knee skin temperature were observed between the TiN-coated CoCrMo TKA (*n* = 51) and a CoCrMo TKA (*n* = 50) [61]. In both groups two knees were revised for reasons unrelated to the TiN-coating, which resulted in a 5-year survival of 96% [61].

## 4. Discussion

In preclinical studies, TiN-coating of implant materials showed to be biocompatible with mainly favorable tribological properties. Several cohort studies of TiN-coated implants showed an overall survival exceeding 90% with a follow-up of 15 to 77 months and good clinical results [55, 58, 62–64]. There were no clinical studies that compared TiN-coated Ti6Al4V implants with uncoated Ti6Al4V implants. One study compared a TiN-coated CoCrMo implant with an uncoated CoCrMo implant and found no difference in clinical outcome or survival [61]. Although preclinical studies showed that TiN-coating of implant material supplies the implant surface with favorable properties, there is insufficient evidence that the TiN-coating affects the clinical outcome and survival of implants in clinical studies. Nonetheless, concerns were raised in a preclinical study about PVD TiN-coated Ti6Al4V because of adhesive coating failure due to coating defects [44]. In retrieved PVD TiN-coated Ti6Al4V femoral heads, the TiN-coating was damaged [53, 54]. Delaminated surface asperities of the TiN-coated femoral head might result in wear debris and lead to adhesive wear on the articular surface [53]. It was suggested that the underlying substrate is prone for third body wear, in case of coating breakthrough [53, 54, 65]. Also, it was advised to handle TiN-coated implants carefully with proper soft instruments, because the thin coating may be easily cracked or scratched by hard surgical tools due to high local stresses at contact points between hard materials opening direct pathways for corrosion leading to delamination [65]. There were no reports of failures of other surface treatments which result in a TiN layer on Ti6Al4V, such as PIRAC [3, 41], PIII [4], and Hardion+ [5].

In a pins-and-plate test of polished PVD TiN-coated CoCrMo a higher surface roughness, catastrophic cohesive failure within the layers of the TiN-coating and a fourfold increase in wear was found [22]. Higher surface roughness of PVD TiN-coated CoCrMo was due to small pits and pinholes related to the PVD process [45]. These pits and pinholes were filled with UHMWPE debris [45, 46]. Although this could be a reason for implant failure, there was no report of failure of a TiN-coated CoCrMo implant in contrast to TiN-coated Ti6Al4V implant. This could be due to a worse TiN-coating layer performance on Ti6Al4V than on CoCrMo [45]. Although the hardness of Ti6Al4V and CoCrMo is similar, Ti6Al4V has a higher difference in elastic modulus with the TiN-coating compared with CoCrMo [45]. It is suggested that this difference in elastic modulus leads to failure of the bonding layer between the TiN-coating and Ti6Al4V [45]. This might induce a cascade of wear and failure of the implant.

A high incidence of aseptic loosening was found with a new design of cemented THA using CoCrMo and TiN-coated titanium heads on UHMWPE liner [52]. There was no significant difference in aseptic loosening of the femoral stem with either the CoCrMo or TiN-coated titanium heads [52]. Furthermore, two brands of cement were used, but no multivariate analysis was performed [52]. Of the cases revised the polyethylene had no visible wear [52]. It remains unclear in this study whether TiN-coated titanium heads have a negative effect on the survival of the implant. That particular implant has been taken off the market.

Prosthetic fractures of the medial flange of size 5 right-sided TiN-coated CoCrMo femoral component of a TKA were found [58, 59]. It is unclear if all size 5 right-sided TiN-coated CoCrMo femoral components in that study fractured. However, the junction of the posterior and the distal chamfer at the medial flange of the implant had a narrow surface cross section, which resulted in a high stress concentration [58]. Adjustments were made to the design and there have been no reports of prosthetic fractures ever since [58]. It is unlikely that the TiN-coating was a cause for these prosthetic fractures.

Noteworthy is that of the 7 clinical studies, case reports not included, 4 studies [55, 62–64] on survival and results of TiN-coated implants were performed by researchers, who patented one of the implants [13] and who also founded the company which produced the implant. This might imply a conflict of interests and might be of influence on the results.

## 5. Conclusion

Titanium alloys used for articulating surfaces require surface treatment to increase hardness and reduce wear. TiN-coating has a favorable effect on the biocompatibility and tribological properties of implant surfaces. However, there are reports of third body wear due to delamination of the PVD TiN-coating on Ti6Al4V, increased UHMWPE wear and cohesive failure of the PVD TiN-coating on CoCrMo of hip implants in preclinical studies, and TiN-coating breakthrough and fretting in a retrieval study of TiN-coated Ti6Al4V femoral heads. These adverse effects might be related to the various coating processes of titanium alloys. The TiN-coating process of titanium alloy articulating surfaces should be optimized and standardized. There were no reports of adverse effects related to TiN-coating of CoCrMo knee implants. Clinical benefit of TiN-coating of CoCrMo knee implant articulating surfaces should be investigated further.

## Figures and Tables

**Table 1:** Preclinical studies on proliferation and differentiation of cells cultured on TiN-coated materials compared with control material.

Study	Cell type	TiN-coated material	Proliferation	Differentiation
van Raay et al. (1995) [20]	Human fibroblasts	Glass cover slips	~	
Groessner-Schreiber et al. (2003) [23]	Mouse fibroblasts	cpTi	+	
Yeung et al. (2007) [4]	Mouse osteoblasts	NiTi; SS; Ti6Al4V	+	
Annunziata et al. (2008) [15]	BMSC	Ti6Al4V	~	~
Annunziata et al. (2011) [16]	BM-MSC	TPS	~	~
Czarnowska et al. (2011) [19]	Saos-2	Ti6Al4V	~	~
Durual et al. (2011) [18]	hOB	cpTi	+	~
Gordin et al. (2012) [5]	hFOB 1.19	cpTi; Ti6Al4V	~	
Rieder et al. (2012) [28]	hOB	cpTi; SS	+	~
van Hove et al. (2013) [27]	MC3T3-E1	CoCrMo	+	~

~: no difference between TiN-coated material and the control; +: higher on TiN-coated material than the control; −: lower on TiN-coated material than the control. TiN: titanium-nitride; BMSC: human bone marrow stromal cells; BM-MSC: human bone marrow mesenchymal stem cells; Saos-2: sarcoma osteogenic, human osteoblast-like cells; hOB: human primary osteoblasts; hFOB 1.19: human fetal-osteoblastic cell line; MC3T3-E1: mouse calvarial osteoblast-like cell; cp Ti: commercially pure titanium; NiTi: nickel-titanium; SS: stainless steel; Ti6Al4V: titanium-aluminum-vanadium alloy; TPS: titanium plasma sprayed.

**Table 2:** Preclinical studies on wear of TiN-coated materials compared with control material.

Study	Test	TiN-coated material	TiN-coating process	Control	Findings	TiN-coating related problems
Coll and Jacquot (1988) [35]	UHMWPE pin on disc	Ti6Al4V	AVID	Ti6Al4V	Reduction of UHMWPE wear^*∗*^	

Maurer et al. (1993) [2]	Screws on plates	Ti6Al4V	PVD/PIII/NII	Ti6Al4V	Reduction of weight loss and metal release^*∗*^	

Venugopalan et al. (2000) [34]	Pin-cylinder scratch test	Ti6Al4V	N	Ti6Al4V	Better scratch resistance^*∗*^	

Hendry and Pilliar (2001) [37]	Fretting test	Ti6Al4V	PVD	Ti6Al4V	No evidence of significant wear^*∗*^	Microparticles and pinholes

Komotori et al. (2001) [44]	Multidirectional scratch testing	Ti6Al4V/SP-700	AIP	TO-Ti6Al4V/SP-700	More mechanical damage^*∗*^	Adhesive coating failure; pitting and blistering

Fisher et al. (2002) [51]	MOM hip simulator	CoCrMo	AEPVD	CoCrMo	Lower wear^*∗*^	Relatively high wear inserts

Williams et al. (2003) [22]	Multidirectional pin on plate	CoCrMo	AEPVD	CoCrMo	Fourfold increase of wear^*∗*^	Cohesive failure

Gutmanas and Gotman (2004) [41]	UHMWPE hip simulator	cpTi/Ti6Al4V	PIRAC	SS	Lower UHMWPE wear rate^*∗*^	

Fisher et al. (2004) [50]	MOM hip simulator	CoCrMo	AEPVD	CoCrMo	Lower wear^*∗*^	Some localized damage

Galvin et al. (2008) [33]	UHMWPE hip simulator	CoCrMo	AEPVD	CoCrMo	Higher UHMWPE volumetric wear rate^*∗*^	

Galetz et al. (2010) [45]	Station-wheel-on-flat testing	CoCrMo	PVD	CoCrMo	No scratches^*∗*^	Polyethylene in pinholes

Kim et al. (2010) [38]	Pin on disc	Ti6Al4V	AIP	Ti6Al4V	Lower wear^*∗*^	Higher wear of TiN compared with TiAlN

Lee et al. (2010) [47]	UHMWPE pin on disc	SS	PIII	CoCrMo	Lower UHMWPE volumetric wear rate^*∗*^	

*∗*: of titanium-nitride coated material compared with the control; TiN: titanium-nitride; UHMWPE: ultrahigh molecular weight polyethylene; MOM: metal-on-metal; SS: stainless steel; Ti6Al4V: titanium-aluminum-vanadium alloy; TO-Ti6Al4V: titanium-oxide coated Ti6Al4V; SP-700: Ti4.5Al3V2Fe2Mo, titanium-aluminum-vanadium-iron-molybdenum alloy; CoCrMo: cobalt-chromium-molybdenum alloy; cp Ti: commercially pure titanium; TiAlN: titanium-aluminum-nitride coating; PIII: plasma immersion ion implantation; AIP: arc ion plating; AVID: arc vapor ion deposition; PVD: physical vapor deposition; NII: nitrogen ion implantation; N: nitrogen diffusion hardening; AEPVD: arc evaporative physical vapor deposition; PIRAC: powder immersion reaction assisted coating.

**Table 3:** Clinical studies on survival of TiN-coated orthopaedic implants.

Study	Type	Prosthesis	TiN-coated material	Number	Fixation	Follow-up	Survival	Reasons for revision
Buechel and Pappas (1992) [64]	Cohort	TAP	Ti6Al4V	14	Cementless	15 (5–24)	100%	None

Massoud et al. (1997) [52]	Cohort	THP	Ti nos	16	Cemented	26	56%	Aseptic loosening

Buechel et al. (2003) [63]	Cohort	TAP	Ti6Al4V	50	Cementless	60 (24–120)	93.5%	Malalignment; PE wear; component subsidence

Buechel et al. (2004) [62]	Cohort	THP	Ti6Al4V	130	Cementless	77 (27–134)	95.5%	Aseptic loosening; bearing dissociation

Buechel and Pappas (2011) [55]	Cohort	RHP	Ti6Al4V	60	Cementless	36 (8–70)	91.8%	Not specified

Mohammed et al. (2014) [58]	Cohort	TKP	CoCrMo	305	Cementless	79 (36–122)	95.1%	Prosthetic fractures; aseptic loosening; alignment

van Hove et al. (2015) [61]	RCT	TKP	CoCrMo	50	Cementless	60	96%	Aseptic loosening

All prosthetic joint articulations were TiN-coated implant material to UHMWPE. Follow-up is presented as mean (range) in months. RCT: randomized clinical trial; TAP: total ankle prosthesis; THP: total hip prosthesis; RHP: resurfacing total hip prosthesis; TKP: total knee prosthesis; Ti6Al4V: titanium-aluminum-vanadium alloy; CoCrMo: cobalt-chromium-molybdenum alloy; Ti nos: titanium not otherwise specified.

## Appendix

Pubmed/Medline Search strategy: (((((((“shoulder”[MeSH Terms] OR “shoulder”[Tiab]) AND (“prosthesis implantation”[MeSH Terms] OR (“prosthesis”[Tiab] AND “implantation”[Tiab]) OR “prosthesis implantation”[Tiab] OR “prosthesis”[Tiab] OR “prostheses and implants”[MeSH Terms] OR (“prostheses”[Tiab] AND “implants”[Tiab]) OR “prostheses and implants”[Tiab])) OR ((“shoulder”[MeSH Terms] OR “shoulder”[Tiab]) AND (“arthroplasty”[MeSH Terms] OR “arthroplasty”[Tiab]))) OR ((“shoulder”[MeSH Terms] OR “shoulder”[Tiab]) AND (“replantation”[MeSH Terms] OR “replantation”[Tiab] OR “replacement”[Tiab]))) OR ((“hip prosthesis”[MeSH Terms] OR (“hip”[Tiab] AND “prosthesis”[Tiab]) OR “hip prosthesis”[Tiab] OR “arthroplasty, replacement, hip”[MeSH Terms] OR (“arthroplasty”[Tiab] AND “replacement”[Tiab] AND “hip”[Tiab]) OR “hip replacement arthroplasty”[Tiab] OR (“hip”[Tiab] AND “prosthesis”[Tiab])) OR ((“hip”[MeSH Terms] OR “hip”[Tiab]) AND (“arthroplasty”[MeSH Terms] OR “arthroplasty”[Tiab])))) OR ((“arthroplasty, replacement, knee”[MeSH Terms] OR (“arthroplasty”[Tiab] AND “replacement”[Tiab] AND “knee”[Tiab]) OR “knee replacement arthroplasty”[Tiab] OR (“knee”[Tiab] AND “arthroplasty”[Tiab]) OR “knee arthroplasty”[Tiab]) OR (“knee prosthesis”[MeSH Terms] OR (“knee”[Tiab] AND “prosthesis”[Tiab]) OR “knee prosthesis”[Tiab] OR “arthroplasty, replacement, knee”[MeSH Terms] OR (“arthroplasty”[Tiab] AND “replacement”[Tiab] AND “knee”[Tiab]) OR “knee replacement arthroplasty”[Tiab] OR (“knee”[Tiab] AND “prosthesis”[Tiab])))) OR ((“ankle”[MeSH Terms] OR “ankle”[Tiab] OR “ankle joint”[MeSH Terms] OR (“ankle”[Tiab] AND “joint”[Tiab]) OR “ankle joint”[Tiab]) AND (“arthroplasty”[MeSH Terms] OR “arthroplasty”[Tiab])) OR (“ankle”[MeSH Terms] OR “ankle”[Tiab] OR “ankle joint”[MeSH Terms] OR (“ankle”[Tiab] AND “joint”[Tiab]) OR “ankle joint”[Tiab]) AND “prosthesis implantation”[MeSH Terms] OR (“prosthesis”[Tiab] AND “implantation”[Tiab]) OR “prosthesis implantation”[Tiab] OR “prosthesis”[Tiab] OR “prostheses and implants”[MeSH Terms] OR “prostheses”[Tiab]) AND ((ti2n[Tiab] OR TiNi[Tiab]) OR (“titanium nitride”[Tiab] AND coating[Tiab])) OR “titanium nitride”[Tiab].

## References

[1] Toth L. E. (1971). *Transition Metal Carbides and Nitrides*.

[2] Maurer A. M., Brown S. A., Payer J. H., Merritt K., Kawalec J. S. (1993). Reduction of fretting corrosion of Ti-6Al-4V by various surface treatments. *Journal of Orthopaedic Research*.

[3] Sovak G., Weiss A., Gotman I. (2000). Osseointegration of Ti6A14V alloy implants coated with titanium nitride by a new method. *The Journal of Bone & Joint Surgery Series B*.

[4] Yeung K. W. K., Poon R. W. Y., Chu P. K. (2007). Surface mechanical properties, corrosion resistance, and cytocompatibility of nitrogen plasma-implanted nickel-titanium alloys: a comparative study with commonly used medical grade materials. *Journal of Biomedical Materials Research Part A*.

[5] Gordin D. M., Gloriant T., Chane-Pane V. (2012). Surface characterization and biocompatibility of titanium alloys implanted with nitrogen by Hardion+ technology. *Journal of Materials Science: Materials in Medicine*.

[6] Dion I., Baquey C., Candelon B., Monties J. R. (1992). Hemocompatibility of titanium nitride. *International Journal of Artificial Organs*.

[7] Sin D.-C., Kei H.-L., Miao X. (2009). Surface coatings for ventricular assist devices. *Expert Review of Medical Devices*.

[8] Schaldach M., Hubmann M., Hardt R., Weikl A. (1989). Titanium nitride cardiac pacemaker electrodes. *Biomedizinische Technik*.

[9] Cogan S. F. (2008). Neural stimulation and recording electrodes. *Annual Review of Biomedical Engineering*.

[10] Mezger P. R., Creugers N. H. J. (1992). Titanium nitride coatings in clinical dentistry. *Journal of Dentistry*.

[11] Wisbey A., Gregson P. J., Tuke M. (1987). Application of PVD TiN coating to Co-Cr-Mo based surgical implants. *Biomaterials*.

[12] Steinemenan S. Implants of titanium or a titanium alloy for the surgical treatment of bones.

[13] Buechel F. F., Pappas M. J. Prosthesis with biologically inert wear resistant surface.

[14] Lombardi A. V., Mallory T. H., Vaughn B. K., Drouillard P. (1989). Aseptic loosening in total hip arthroplasty secondary to osteolysis induced by wear debris from titanium-alloy modular femoral heads. *Journal of Bone and Joint Surgery—American Volume*.

[15] Annunziata M., Guida L., Perillo L., Aversa R., Passaro I., Oliva A. (2008). Biological response of human bone marrow stromal cells to sandblasted titanium nitride-coated implant surfaces. *Journal of Materials Science: Materials in Medicine*.

[16] Annunziata M., Oliva A., Basile M. A. (2011). The effects of titanium nitride-coating on the topographic and biological features of TPS implant surfaces. *Journal of Dentistry*.

[17] Manso-Silvan M., Martínez-Duart J. M., Ogueta S., García-Ruiz P., Pérez-Rigueiro J. (2002). Development of human mesenchymal stem cells on DC sputtered titanium nitride thin films. *Journal of Materials Science: Materials in Medicine*.

[18] Durual S., Pernet F., Rieder P., Mekki M., Cattani-Lorente M., Wiskott H. W. A. (2011). Titanium nitride oxide coating on rough titanium stimulates the proliferation of human primary osteoblasts. *Clinical Oral Implants Research*.

[19] Czarnowska E., Morgiel J., Ossowski M., Major R., Sowinska A., Wierzchon T. (2011). Microstructure and biocompatibility of titanium oxides produced on nitrided surface layer under glow discharge conditions. *Journal of Nanoscience and Nanotechnology*.

[20] van Raay J. J. A. M., Rozing P. M., van Blitterswijk C. A., van Haastert R. M., Koerten H. K. (1995). Biocompatibility of wear-resistant coatings in orthopaedic surgery in vitro testing with human fibroblast cell cultures. *Journal of Materials Science: Materials in Medicine*.

[21] Yeniyol S., Bölükbaşı N., Bilir A., Çakır A. F., Yeniyol M., Ozdemir T. (2013). Relative contributions of surface roughness and crystalline structure to the biocompatibility of titanium nitride and titanium oxide coatings deposited by PVD and TPS coatings. *ISRN Biomaterials*.

[22] Williams S., Tipper J. L., Ingham E., Stone M. H., Fisher J. (2003). In vitro analysis of the wear, wear debris and biological activity of surface-engineered coatings for use in metal-on-metal total hip replacements. *Proceedings of the Institution of Mechanical Engineers, Part H: Journal of Engineering in Medicine*.

[23] Groessner-Schreiber B., Neubert A., Müller W.-D., Hopp M., Griepentrog M., Lange K.-P. (2003). Fibroblast growth on surface-modified dental implants: an in vitro study. *Journal of Biomedical Materials Research, Part A*.

[24] Serro A. P., Completo C., Colaço R. (2009). A comparative study of titanium nitrides, TiN, TiNbN and TiCN, as coatings for biomedical applications. *Surface and Coatings Technology*.

[25] Cyster L. A., Parker K. G., Parker T. L., Grant D. M. (2003). The effect of surface chemistry and nanotopography of titanium nitride (TiN) films on 3T3-L1 fibroblasts. *Journal of Biomedical Materials Research. Part A*.

[26] Cho D. R., Shanbhag A. S., Hong C.-Y., Baran G. R., Goldring S. R. (2002). The role of adsorbed endotoxin in particle-induced stimulation of cytokine release. *Journal of Orthopaedic Research*.

[27] van Hove R. P., Nolte P. A., Semeins C. M., Klein-Nulend J. (2013). Differences in proliferation, differentiation, and cytokine production by bone cells seeded on titanium-nitride and cobalt-chromium-molybdenum surfaces. *Journal of Biomaterials Applications*.

[28] Rieder P., Scherrer S., Filieri A., Wiskott H. W. A., Durual S. (2012). TiNOx coatings increase human primary osteoblasts proliferation independently of the substrate: a short report. *Bio-Medical Materials and Engineering*.

[29] Clem W. C., Konovalov V. V., Chowdhury S., Vohra Y. K., Catledge S. A., Bellis S. L. (2006). Mesenchymal stem cell adhesion and spreading on microwave plasma-nitrided titanium alloy. *Journal of Biomedical Materials Research Part A*.

[30] Catledge S. A., Vohra Y. K., Bellis S. L., Sawyer A. A. (2004). Mesenchymal stem cell adhesion and spreading on nanostructured biomaterials. *Journal of Nanoscience and Nanotechnology*.

[31] Hayashi K., Matsuguchi N., Uenoyama K., Kanemaru T., Sugioka Y. (1989). Evaluation of metal implants coated with several types of ceramics as biomaterials. *Journal of Biomedical Materials Research*.

[32] Larsson Wexell C., Thomsen P., Aronsson B.-O. (2013). Bone response to surface-modified titanium implants: studies on the early tissue response to implants with different surface characteristics. *International Journal of Biomaterials*.

[33] Galvin A., Brockett C., Williams S. (2008). Comparison of wear of ultra-high molecular weight polyethylene acetabular cups against surface-engineered femoral heads. *Proceedings of the Institution of Mechanical Engineers, Part H: Journal of Engineering in Medicine*.

[34] Venugopalan R., Weimer J. J., George M. A., Lucas L. C. (2000). The effect of nitrogen diffusion hardening on the surface chemistry and scratch resistance of Ti-6Al-4V alloy. *Biomaterials*.

[35] Coll B. F., Jacquot P. (1988). Surface modification of medical implants and surgical devices using TiN layers. *Surface and Coatings Technology*.

[36] Goldberg J. R., Gilbert J. L. (2003). In vitro corrosion testing of modular hip tapers. *Journal of Biomedical Materials Research—Part B Applied Biomaterials*.

[37] Hendry J. A., Pilliar R. M. (2001). The fretting corrosion resistance of PVD surface-modified orthopedic implant alloys. *Journal of Biomedical Materials Research*.

[38] Kim H., Kim C. Y., Kim D. W. (2010). Wear performance of self-mating contact pairs of TiN and TiAlN coatings on orthopedic grade Ti-6Al-4V. *Biomedical Materials*.

[39] Starosvetsky D., Shenhar A., Gotman I. (2001). Corrosion behavior of PIRAC nitrided Ti-6Al-4V surgical alloy. *Journal of Materials Science: Materials in Medicine*.

[40] Rodríguez D., Manero J. M., Gil F. J., Planell J. A. (2001). Low cycle fatigue behavior of Ti6AI4V thermochemically nitrided for its use in hip prostheses. *Journal of Materials Science: Materials in Medicine*.

[41] Gutmanas E. Y., Gotman I. (2004). PIRAC Ti nitride coated Ti-6AI-4V head against UHMWPE acetabular cup-hip wear simulator study. *Journal of Materials Science: Materials in Medicine*.

[42] Pappas M. J., Makris G., Buechel F. F. (1995). Titanium nitride ceramic film against polyethylene. A 48 million cycle wear test. *Clinical Orthopaedics and Related Research*.

[43] Peterson C. D., Hillberry B. M., Heck D. A. (1988). Component wear of total knee prostheses using Ti-6Al-4V, titanium nitride coated Ti-6Al-4V, and cobalt-chromium-molybdenum femoral components. *Journal of Biomedical Materials Research*.

[44] Komotori J., Lee B. J., Dong H., Dearnley P. A. (2001). Corrosion response of surface engineered titanium alloys damaged by prior abrasion. *Wear*.

[45] Galetz M. C., Fleischmann E. W., Konrad C. H., Schuetz A., Glatzel U. (2010). Abrasion resistance of oxidized zirconium in comparison with CoCrMo and titanium nitride coatings for artificial knee joints. *Journal of Biomedical Materials Research, Part B, Applied Biomaterials*.

[46] Galetz M. C., Seiferth S. H., Theile B., Glatzel U. (2010). Potential for adhesive wear in friction couples of UHMWPE running against oxidized zirconium, titanium nitride coatings, and cobalt-chromium alloys. *Journal of Biomedical Materials Research Part B: Applied Biomaterials*.

[47] Lee S. B., Choi J. Y., Park W. W. (2010). A study of TiN-coated metal-on-polymer bearing materials for hip prosthesis. *Metals and Materials International*.

[48] Jones V. C., Auger D. D., Stone M. H., Fisher J. (2002). New materials for mobile bearing knee prosthesis—titanium nitride counterface coatings for reduction of polyethylene wear. *LCS Mobile Bearing Knee Arthroplasty*.

[49] Gispert M. P., Serro A. P., Colaço R., Pires E., Saramago B. (2008). The effect of roughness on the tribological behavior of the prosthetic pair UHMWPE/TiN-coated stainless steel. *Journal of Biomedical Materials Research, Part B, Applied Biomaterials*.

[50] Fisher J., Hu X. Q., Stewart T. D. (2004). Wear of surface engineered metal-on-metal hip prostheses. *Journal of Materials Science: Materials in Medicine*.

[51] Fisher J., Hu X. Q., Tipper J. L. (2002). An *in vitro* study of the reduction in wear of metal-on-metal hip prostheses using surface-engineered femoral heads. *Proceedings of the Institution of Mechanical Engineers, Part H: Journal of Engineering in Medicine*.

[52] Massoud S. N., Hunter J. B., Holdsworth B. J., Wallace W. A., Juliusson R. (1997). Early femoral loosening in one design of cemented hip replacement. *The Journal of Bone and Joint Surgery—British Volume*.

[53] Harman M. K., Banks S. A., Andrew Hodge W. (1997). Wear analysis of a retrieved hip implant with titanium nitride coating. *Journal of Arthroplasty*.

[54] Raimondi M. T., Pietrabissa R. (2000). The in-vivo wear performance of prosthetic femoral heads with titanium nitride coating. *Biomaterials*.

[55] Buechel F. F., Pappas M. J. (2011). A metal/ultrahigh-molecular-weight polyethylene cementless surface replacement. *Seminars in Arthroplasty*.

[56] Malviya A., Lobaz S., Holland J. (2007). Mechanism of failure eleven years following a Buechel Pappas hip resurfacing. *Acta Orthopaedica Belgica*.

[57] Morscher E., Bereiter H., Lampert C. (1989). Cementless press-fit cup. Principles, experimental data, and three-year follow-up study. *Clinical Orthopaedics and Related Research*.

[58] Mohammed A., Metcalfe A., Woodnutt D. (2014). Medium-term outcome of titanium nitride, mobile bearing total knee replacement. *Acta Orthopaedica Belgica*.

[59] Könst Y. E., Posthuma De Boer J., Saouti R. (2010). Fracture of a ceramic coated implant total knee prosthesis. *Nederlands Tijdschrift voor Orthopaedie*.

[60] Park S., Kim H., In Y. (2014). Fracture of titanium nitride-coated femoral component after total knee arthroplasty. *The Knee*.

[61] van Hove R. P., Brohet R. M., van Royen B. J., Nolte P. A. (2015). No clinical benefit of titanium nitride coating in cementless mobile-bearing total knee arthroplasty. *Knee Surgery, Sports Traumatology, Arthroscopy*.

[62] Buechel F. F., Buechel F. F., Helbig T. E., D'Alessio J., Pappas M. J. (2004). Two- to 12-year evaluation of cementless Buechel-Pappas total hip arthroplasty. *The Journal of Arthroplasty*.

[63] Buechel F. F., Buechel F. F., Pappas M. J. (2003). Ten-year evaluation of cementless Buechel-Pappas meniscal bearing total ankle replacement. *Foot and Ankle International*.

[64] Buechel F. F., Pappas M. J. (1992). Survivorship and clinical evaluation of cementless, meniscal-bearing total ankle replacements. *Seminars in Arthroplasty*.

[65] Lappalainen R., Santavirta S. S. (2005). Potential of coatings in total hip replacement. *Clinical Orthopaedics and Related Research*.

